# Analysis of Low Frequency Protein Truncating Stop-Codon Variants and Fasting Concentration of Growth Hormone

**DOI:** 10.1371/journal.pone.0128348

**Published:** 2015-06-18

**Authors:** Erik Hallengren, Peter Almgren, Gunnar Engström, Margaretha Persson, Olle Melander

**Affiliations:** 1 Department of Clinical Sciences, Lund University, Malmö, Sweden; 2 Department of Internal Medicine, Skåne University Hospital, Malmö, Sweden; National Cancer Institute, National Institutes of Health, UNITED STATES

## Abstract

**Background:**

The genetic background of Growth Hormone (GH) secretion is not well understood. Mutations giving rise to a stop codon have a high likelihood of affecting protein function.

**Objectives:**

To analyze likely functional stop codon mutations that are associated with fasting plasma concentration of Growth Hormone.

**Methods:**

We analyzed stop codon mutations in 5451 individuals in the Malmö Diet and Cancer study by genotyping the Illumina Exome Chip. To enrich for stop codon mutations with likely functional effects on protein function, we focused on those disrupting >80% of the predicted amino acid sequence, which were carried by ≥10 individuals. Such mutations were related to GH concentration, measured with a high sensitivity assay (hs-GH) and, if nominally significant, to GH related phenotypes, using linear regression analysis.

**Results:**

Two stop codon mutations were associated with the fasting concentration of hs-GH. rs121909305 (NP_005370.1:p.R93*) [Minor Allele Frequency (MAF) = 0.8%] in the Myosin 1A gene (*MYO1A*) was associated with a 0.36 (95%CI, 0.04 to 0.54; p=0.02) increment of the standardized value of the natural logarithm of hs-GH per 1 minor allele and rs35699176 (NP_067040.1:p.Q100*) in the Zink Finger protein 77 gene (*ZNF77*) (MAF = 4.8%) was associated with a 0.12 (95%CI, 0.02 to 0.22; p = 0.02) increase of hs-GH. The mutated high hs-GH associated allele of *MYO1A* was related to lower BMI (β-coefficient, -0.22; p = 0.05), waist (β-coefficient, -0.22; p = 0.04), body fat percentage (β-coefficient, -0.23; p = 0.03) and with higher HDL (β-coefficient, 0.23; p = 0.04). The ZNF77 stop codon was associated with height (β-coefficient, 0.11; p = 0.02) but not with cardiometabolic risk factors.

**Conclusion:**

We here suggest that a stop codon of *MYO1A*, disrupting 91% of the predicted amino acid sequence, is associated with higher hs-GH and GH-related traits suggesting that *MYO1A* is involved in GH metabolism and possibly body fat distribution. However, our results are preliminary and need replication in independent populations.

## Introduction

Growth Hormone (GH) is secreted from the anterior pituitary gland and exerts important biological actions throughout the entire life, such as protein anabolism in muscles, lipolysis in adipose tissue and regulation of glucose and lipid metabolism [1]. In healthy individuals lower values of basal GH are associated with higher values of body mass index (BMI), waist, low density lipoprotein cholesterol (LDL-C) and lower values of high density lipoprotein cholesterol (HDL-C) [2–4]. With these associations indicating a more adverse cardiovascular risk profile one might assume that GH is beneficial, however our recent results contradict this, showing that higher fasting levels of GH are associated with increased cardiovascular morbidity and mortality [2], confirming similar previous results from *Maison et al* [5]. With these results it would be interesting to find genetic determinants of GH and in a later phase investigate their relation to cardiovascular disease. As mentioned in our previous article [2], GH plasma concentration is relatively stable in the morning hours [6–8] and we used a high sensitivity assay for plasma GH measurements (hs-GH) able to quantify even the low normal range of GH-levels.

Genome-wide association studies (GWAS) have seen a large increase in the last few years and in 2,111 publications over 15 000 (February 2015) common single nucleotide polymorphisms (SNP) associated with complex human traits and diseases have been found [9]. Despite this tremendous amount of successful studies, a lot of the presumed heritability is still unaccounted for and it is becoming evident that GWAS targeting common genetic variants are insufficient to explain a lot of the missing heritability [10–12]. For example BMI is a polygenetic trait believed to be heritable to a degree of above 40% [11]. A recent GWAS and metabochip analysis of over 339,000 individuals found 97 loci associated with BMI, but these only explained ~3% of the phenotypic variance [10]. It is believed that the common snps genotyped today could at the most explain roughly 50% of the heritability (i.e. 20% of phenotypic variance) of BMI [10,11]. Figures for other traits such as LDL-C, HDL-C and height are roughly the same, with common snps supposed to explain 50–60% of the heritability with a theoretically infinite sample size [11,13]. Empirically, monogenetic diseases are very rare but the underlying mutations are characterized by high phenotypic penetrance. Thus, it has been suggested that low frequency and rare variants (present in <5% and <1% of the population, respectively) may have large effect sizes that cannot fully be captured in traditional GWAS as the latter is designed to identify predominantly common gene variants [12,14,15], and that such low frequency and rare variants may explain part of the missing heritability. Although studies of such variants have shown larger effect sizes compared to GWAS [16–18], a larger part of the heritability still remains to be explained.

The exome chip is a chip-based array including approximately 250,000 previously identified coding variants [including missense variants, nonsense (stop codon) variants and splice site variants], the majority of which occur at low frequency (<5%) [19,20]. Most efforts to analyze the plethora of coding variants of the exome chip, have been performed using candidate gene approaches or burden tests where the latter assume that all mutations at a locus functionally affect the trait of interest [16,17,21]. This assumption is unlikely to be true and thus lead to false negative results if only one out of the many variants at a locus is in fact functional and affect the phenotype of interest [22].

We here genotyped a cohort of 5451 individuals using an exome chip and in an attempt to filter out the variants most likely to be of functional importance, focused on stop codon mutations which disrupt at least 80% of the predicted amino acid sequence of the encoded protein. Using this strategy we aimed at identifying stop codon mutations that affect fasting GH concentration, and possibly GH related phenotypes.

Specifically, we applied the following criteria: The mutation should encode a stop codon, disrupt at least 80% of the protein, have at least 10 carriers in our sample, minor allele frequency (MAF)<0.05 and a p-value of <0.05 in a linear regression model with GH.

## Methods

The Malmö diet and cancer study (MDC) is a population-based, prospective cohort of males born between 1923 and 1945 and women born between 1923 and 1950. A total of 28 449 individuals were examined between 1991 and 1996 [23]. From this cohort a random sample, examined between November 1991 and February 1994 (n = 6103) were included in the MDC cardiovascular cohort (MDC-CC), with the primary aim to study the epidemiology of carotid artery disease [24]. The primary study cohort consists of those in which DNA was available and genotyping of the exome chip was successful (n = 5451), i.e. past our QC criteria.

All participants provided written consent and the study was approved by Regional Ethical Review Board in Lund, Sweden.

At baseline (1991–96) participants underwent a medical history, physical examination and laboratory assessment. Levels of HDL-C and total cholesterol were measured according to standard procedures at the Department of Clinical Chemistry, University Hospital of Malmö. The levels of LDL-C were calculated according to the Friedewald formula.

All samples of plasma and whole-blood were obtained after overnight fasting and samples were drawn between 7.30 am and 9.00 am. Further details about the clinical examination can be found in previous articles [2,23,24].

GH levels were measured in stored fasting plasma samples, which had been frozen to -80°C immediately at the MDC-CC baseline examination. The measurement was made with a high-sensitivity chemiluminescence sandwich immunoassay previously described (SphingoTec GmbH, Borgsdorf, Germany) [2]. Individuals with GH-value equal to 0 (n = 12) were censored in the analyses including GH as a variable.

Genotyping of the cohort was made using the HumanOmniExpressBeadChip and iScan system (Illumina, San Diego, CA, USA) analyzing 250 000 coding variants (missense variants, nonsense variants and splice site variants).

A multivariate linear regression model with log-transformed hs-GH as the dependent variable were executed for each of the stop codons and adjusted for gender and age using PLINK software [25]. The variants that fulfilled all of the following criteria were then selected for further analyses:

- MAF < 5%. In order not to catch variants that has been captured in previous GWAS-assays.
- At least 10 carriers of the allele in our sample (= minor allele frequency (MAF) ≥ 0.1%) to exclude mutations that are so rare that statistical analysis is not possible.
- Nonsense mutation, i. e. the variant encodes a stop codon instead of an amino acid, disrupting the protein with possibly loss-of-function for the gene. The UCSC genome browser (UCSC Genome Browser on Human Feb. 2009 (GRCh37/hg19 Assembly, http://genome.ucsc.edu/)) [26] was used to confirm the location and genetic code.
- The mutation disrupts at least 80% of the protein, for example amino acid (AA) number 30 out of 289 is replaced by a stop codon. Thereby making the chances for biological relevance greater. The Uniprot Knowledgebase (http://www.uniprot.org) [27] was used as reference for the number of AA in the protein. If multiple splice variants were known to exist for the protein, this criterion had to be fulfilled in all splice variants available in the CCDS-database (Consensus coding sequences; [http://www.ncbi.nlm.nih.gov/CCDS/CcdsBrowse.cgi]) [28,29].
- *P*<0.05 in the linear regression model for the association between the stop codon mutation and GH.

**Fig 1:**
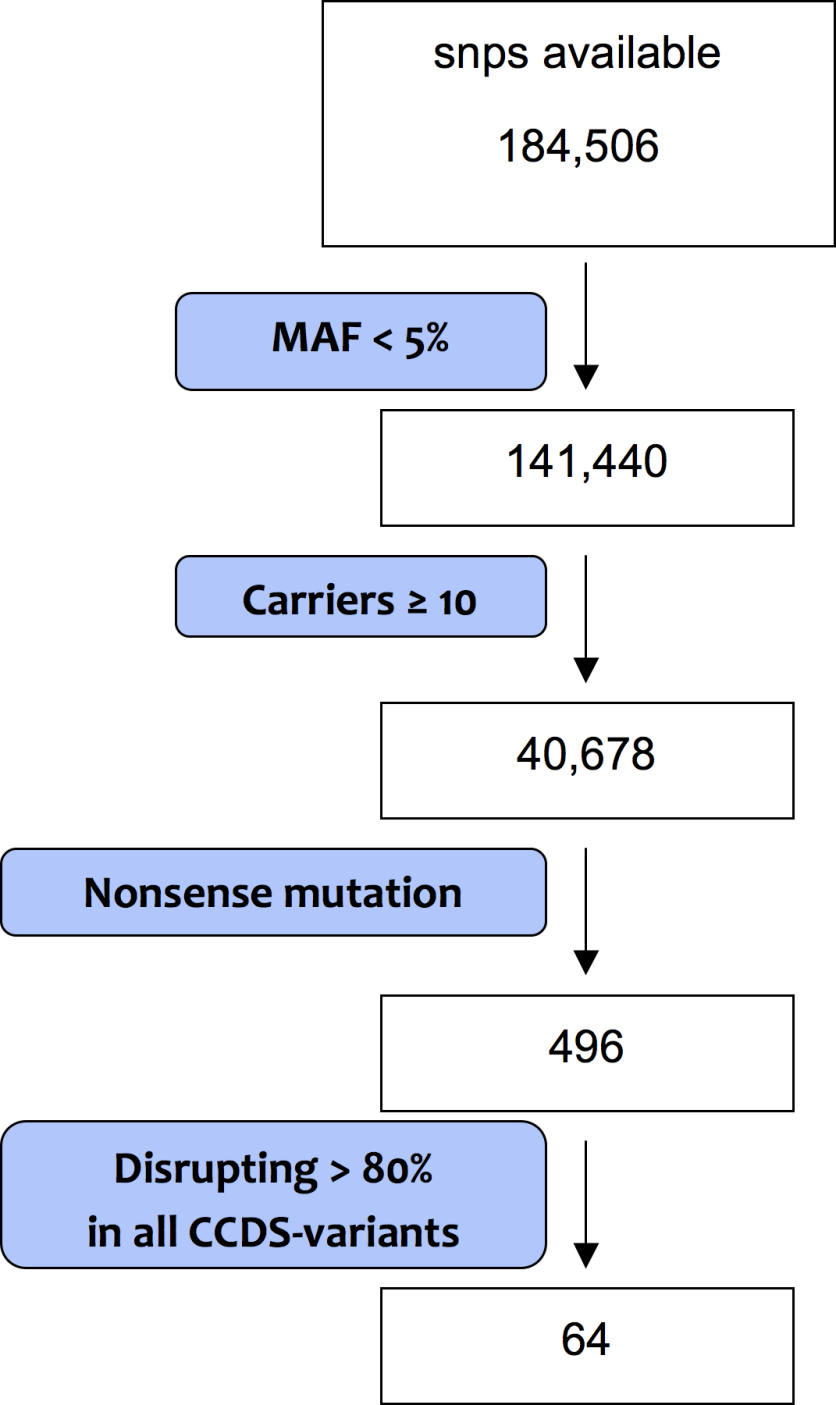
Selection of snps. Numbers indicated number of snp available in each step. Detailed selection criteria can be found in the methods section.

The stop codon mutations that fulfilled all of the above criteria were selected for further analysis. The number of snps available in each step is shown in Fig 1.

The following analyses were performed in SPSS (version 22.0.0, SPSS Inc, Chicago, Ill). A 2-sided P value of less than 0.05 was considered nominally significant.

We used variables known to be associated with fasting hs-GH from our previous study [2]. These were LDL-C, HDL-C, BMI, waist and body fat percentage. Height was also added since we hypothesized that it would correlate with GH. Given the difference in fasting GH concentration between the genders the variables including hs-GH were transformed into the natural logarithm and standardized separately for men and women before being used in any analysis. Multivariate regression models adjusted for sex and age were then analyzed between GH and the various variables stated above.

After selection of the stop codon variants as described above, these variants were analyzed vs the GH-associated traits in order to test if any genetic effect on hs-GH may translate into an effect on GH-related traits. Each of the GH-associated variables were entered as the dependent variable into multivariate regression models with sex, age and one stop codon mutation per model. We assumed an additive model of inheritance in all analyses. If the association snp vs GH-related trait was nominally significant we performed a mediation analysis to test whether the effect of the snp on the trait was mediated through hs-GH [30].

## Results

Baseline characteristics of the participants are shown in Table 1. The individuals in whom genotyping could not be performed due to missing samples or poor call rates in the genotyping did not differ in their baseline characteristics (S1 Table).

**Table 1:** Clinical characteristics of the study population.

Variable		Participants with data
Female (%)	3187 (58.5)	5451
Age, mean (SD), years	57.5 (5.9)	5451
Body Mass Index, Mean (SD), kg/m2	25.8 (4.0)	5444
Waist, mean (SD), cm	84.1 (13.0)	5443
Bodyfat percentage, mean (SD), %	27.6 (7.1)	5434
LDL-C, mean (SD), mmol/L	4.20 (0.98)	4800
HDL-C, mean (SD), mmol/L	1.38 (0.37)	4874
Height, mean (SD), cm	169 (9)	5444
Growth Hormone—males, median (IQR), μg/L	0.11 (0.06–0.33)	1729
Growth Hormone—females, median (IQR), μg/L	1.21 (0.38–3.12)	2405

LDL-C, Low-density lipoprotein cholesterol; HDL-C, High-density lipoprotein cholesterol

In the multivariate adjusted linear regression models hs-GH displayed nominally significant negative associations with BMI, waist and LDL-C and nominally significant positive ones with HDL-C and height (Table 2). The R^2^ was highest in the analysis for waist (6,8%) and lowest for height (0,5%).

**Table 2:** Results from multiple linear regression models adjusted for sex and age examining correlations between fasting values of growth hormone and traditionally associated variables.

Variable	β coefficient*	95% CI	*P*
BMI^†^	-0.24	-0.27 to -0.21	6.65E-56
Waist	-0.25	-0.28 to -0.22	1.72E-58
Bodyfat percentage	-0.23	-0.26 to -0.20	6.15E-51
HDL-C^†^	0.19	0.16 to 0.22	1.02E-34
LDL-C^†^	-0.13	-0.16 to -0.10	1.11E-15
Height	0.04	0.01 to 0.07	0.007

One regression model is performed per variable with hs-GH as the dependent variable vs the variable in question, sex and age as independent variables.

* The β coefficients are expressed as the increment of standardized values of the natural logarithm of hs-GH per 1 increment of standardized values of the natural logarithm of the variable in question. Variables and hs-GH are standardized separately in men and women before being used in the combined analysis.

†Abbreviations: BMI, Body Mass Index; LDL-C, Low-density lipoprotein cholesterol; HDL-C, High-density lipoprotein cholesterol

We identified 2 stop codon mutations, fulfilling the criteria set up, with nominally significant association with hs-GH, one in the Myosin 1A gene (*MYO1A*) and one in the Zink Finger Protein 77 gene (*ZNF77*) (Table 3). Illumina cluster plots indicated that the genotyping was of good quality (S1 and S2 Figs). A total of 64 snps fulfilled the criteria set up previous to the association test with hs-GH (S2 Table). The stop codon of *MYO1A* (rs121909305), which occurred at a MAF of 0.8%, replaces an arginine at amino acid position 93 with a premature stop codon (NP_005370.1:p.R93*), thereby deleting 91% of the predicted *MYO1A* amino acid sequence. The stop codon of *ZNF77* (rs35699176), occurred at a MAF of 4.8% and replaces a glutamine residue (NP_067040.1:p.Q100*) resulting in deletion of 82% of the predicted *ZNF77* amino acid sequence. If the cut-off had been set at 50%, 2 additional stop codon variants had been added.

**Table 3:** Stop codon mutations fulfilling the criteria previously described.*

Stop codon mutation							Relation to hs-GH		
snp	Gene	Mutation†	Protein length‡	Carriers—total§	Carriers—GH¶	MAF#	Beta**	95%CI	*P*
rs35699176	*ZNF77*	p.Q100*	545	17/493/4940	13/364/3756	4.8%	0.12	0.02 to 0.22	0.02
rs121909305	*MYO1A*	p.R93*	1043	0/86/5365	0/63/4071	0,8%	0.36	0.04 to 0.54	0.02

*MAF < 5%, mutation giving rise to stop codon, >9 carriers in our sample, nominally significant association with hs-GH (*P*<0.05), mutation disrupting > 80% of the protein and present in all splice variants.

†Original amino acid, amino acid no

‡Number of amino acids in the predicted amino acid sequence

§Number of homozygous carriers for minor allele, heterozygous and homozygous carriers for major allele respectively

¶Number of carriers in which values of hs-GH were available

#Minor allele frequency in our sample

**The β coefficients are expressed as the increment of standardized values of the natural logarithm of hs-GH per 1 minor allele. NB hs-GH is standardized separately in men and women before being used in the combined analysis.

“Relation to hs-GH” shows the association from linear regression models adjusted for sex and age.

Presence of the minor allele was in both stop codon mutations associated with higher hs-GH (Table 3). In our material there were individuals who were both heterozygous and homozygous for the minor allele in rs35699176 (*ZNF77*), while no individuals were homozygous for the minor allele in rs121909305 (*MYO1A*).

To test if the effect of the identified stop codon mutations on hs-GH may influence GH-related traits they were related to anthropometric measures and lipoprotein levels to which we found significant relationships for fasting concentration of plasma hs-GH. The results of the linear regression models, adjusted for gender and age are shown in Table 4. The mutated high hs-GH associated allele of *MYO1A* was nominally related to lower BMI, waist, body fat percentage and with higher HDL. The *ZNF77* stop codon variant related to higher hs-GH was positively nominally associated with height but not with anthropometric or lipoprotein traits. A mediation analysis testing the previous mentioned nominally significant traits resulted in nominal significance for hs-GH and non-significant results for the snps in all traits suggesting that the association between the snps and traits were mediated through hs-GH (S3 Table).

**Table 4:** Linear regression models adjusted for sex and age exploring the relation between various GH-associated variables and the stop codon mutations.

SNP	Variable	Beta*	95%CI	*P*
rs35699176	BMI^†^	-0.033	-0.120 to 0.053	0.45
(*ZNF77*)	Bodyfat%	-0.063	-0.149 to 0.023	0.15
	Waist	0.005	-0.081 to 0.091	0.91
	LDL^†^	0.009	-0.082 to 0.101	0.84
	HDL^†^	-0.012	-0.105 to 0.080	0.80
	Height	0.106	0.020 to 0.191	0.02
rs121909305	BMI^†^	-0.216	-0.428 to -0.004	0.05
(*MYO1A*)	Bodyfat%	-0.234	-0.445 to -0.024	0.03
	Waist	-0.219	-0.432 to -0.006	0.04
	LDL^†^	-0.180	-0.403 to 0.043	0.11
	HDL^†^	0.234	0.009 to 0.459	0.04
	Height	0.020	-0.190 to 0.230	0.85

One model is executed per variable with the variable in question as the dependent variable and the stop codon mutation, sex and age as independent variables.

*The β coefficients are expressed as the increment of standardized values of the natural logarithm of the variable per presence of 1 minor allele. The variables are standardized separately in men and women before being used in the combined analysis.

†Abbreviations: BMI, Body Mass Index; LDL-C, Low-density lipoprotein cholesterol; HDL-C, High-density lipoprotein cholesterol

## Discussion

We here focused exome chip analysis to variants resulting in premature stop codons disrupting the majority of the protein, thereby being likely to alter protein function. In addition, we restricted the search to low-frequency variants (MAF<5%), making them less likely to having been picked up by previous GWAS analyses but at the same time not being so rare that they would be impossible to statistically handle (at least 10 carriers, here corresponding to a MAF>0.1%). Thereby we identified a premature stop codon in *MYO1A* associated with higher hs-GH and lower measures of obesity as well as higher levels of HDL-C and one premature stop codon of *ZNF77* associated with higher hs-GH and height. Given the large proportions of the predicted amino acid sequences of these two proteins which were deleted as a consequence of the stop codon variants (91 and 82%, respectively), it is reasonable to assume that the function of the two proteins would be reduced, rather than enhanced, however, the knowledge of active sites and regulatory domains of the two proteins are limited, so gain of function as a result of the mutations cannot be excluded.

Although there have been reports on genetic variants in genes coding for components of the GH-system which are associated with GH-related traits, e.g. genetic variance of the GH receptor[31], studies on genetic determinants in relation to fasting GH concentration are rare. We are only aware of one previous article, in which a SNP in *GH1* was found to be associated with the fasting concentration of GH [32]. Our result thus encourages not only replication efforts of the two mutations in relation to fasting GH concentration but also studies in much larger cohorts to test if the genetic elevation of GH mediated by stop codons of *MYO1A* and *ZNF77* could be replicated and if it would be associated with risk of cardiovascular disease and mortality.

The stop codon mutation in *MYO1A* was interesting in the sense that it showed associations both to GH and to GH-related phenotypes. The minor allele was associated with an increase in fasting plasma GH and affected the GH-related variables as could be expected, thereby strengthening the hypothesis that it could be a genetic determinant of fasting plasma GH. A mediation analysis indicated that the effect of the mutated allele on the GH-related variables, were mediated through hs-GH and not from pleiotropic effects. Although the results are exciting, the multiple testing is an issue that requires cautious interpretation of the results. *MYO1A* is an unconventional myosin located on 12q13.3 and the very same stop codon mutation (rs121909305) was suggested to be responsible for autosomal dominant hearing loss in 2003 [33]. However knockout models in mice and a recent GWAS did not confirm this association and the authors in the latter concluded that *MYO1A* seemed “dispensable for hearing and overall non-essential” [34,35]. In the knockout mice the *MYO1A* protein could be identified in enterocytes and was found to be important for the composition and regulation of the brush border. However the mice did not exhibit any overt phenotype and growth and weight gain were similar to wild-type littermates [34]. The study did not focus on GH-function and it would be interesting to study the KO-mice from this perspective, with more thorough measurements of body composition and observations of life span, since the function of the GH-axis has been shown to affect life span in mice [36–38]. To our knowledge, our study is the first suggesting a connection between GH and *MYO1A*.

The other stop codon mutation that passed all the criteria was rs35699176, present in *ZNF77*, a zinc finger protein located on chromosome 19p13.3 and containing four exons encoding a 545 amino acid protein [26,27,39]. In the analyses with GH-associated phenotypes, the only association was with height, which is not one of the stronger associations with hs-GH in adults (Table 2). The variant is relatively common with a MAF of 4.8%, just passing our threshold of <0.05. This probably makes it common enough to be detectable in a GWAS and thus makes it less attractable to analyze with our approach.

Publications on this gene are scarce. One whole exome sequencing study from 2013 examining patients with fibromyalgia found that this exome variant (rs35699176) was associated with fibromyalgia based on the inheritance pattern from their parents and elevated levels of the inflammatory cytokine, IL-12 [40]. GH has previously been connected to the function of the immune system and a study of short stature non-GHD children showed that administration of GH increased levels of IL-12 [41]. A subsequent study from the same research group, examining children *with* GHD did not show the same effect of GH administration [42]. Thus an effect of ZNF77 on GH metabolism is a possibility, however more studies investigating this relationship are required.

Our study has several limitations. Since we are using a new approach for analyzing data from the exome chip, the method is not previously validated. We have tried to apply stringent criteria that would be easy to follow and strived to find biologically relevant mutations. By finding association not only between the stop codon mutations and GH, but also between the mutations and a GH-related phenotype our findings have larger credibility. However they need to be replicated in a larger cohort, especially since the stop codon mutations are rare. It may be that with a similar analysis and larger sample sizes one might find additional genetic determinants of plasma GH.

The cut-off at 80% is not based on any biological finding but rather follows the logic that an early truncation ought to have a larger effect on the protein. If this had been set to 50%, 2 additional stop codon mutations had been included.

We have used p<0.05 as significance level and it is a risk that we due to multiple testing have encountered false positives. 64 stop codon mutations were analyzed vs hs-GH. A Bonferroni corrected p-value would be 0.05/64 = 0.00078. None of the variants fulfill this and a larger study is probably necessary to achieve this significance level. This underlines the need of replicating our results in other populations.

As we pointed out in our previous study [2], the use of fasting hs-GH is not a standard clinical test and the release of GH in pulses complicates measurement. However as mentioned earlier, measurements were made in the morning when plasma GH concentration is relatively stable [6–8]. The amount of non-accepters to the initial invitation was quite large, which could make a bias selection of a more healthy population. There was no suspicion of selection bias in people not included in the genetic analysis.

If our result with the stop codon mutations in *MYO1A* and/or *ZNF77* being genetic determinants of plasma GH could be replicated in another study it would be interesting to further investigate if these variants are associated with an increased cardiovascular morbidity and mortality as previously suggested [2,5]. Unfortunately the current study is underpowered to answer this question.

In conclusion we here demonstrate a new way to analyze rare genetic variants from the exome chip and find that a stop codon mutation in *MYO1A* (rs121909305) is nominally associated to the fasting level of plasma GH. A stop codon mutation in ZNF77 was also nominally associated to fasting plasma GH but the connection to a GH-related phenotype was weaker. Our findings need to be replicated in a larger cohort, which with morbidity data could provide new insights regarding GH and cardiovascular diseases.

## Supporting Information

S1 Table Clinical characteristics of the study population and individuals in which genotyping was not possible due to missing samples or unsuccessful genotyping. Abbreviations: LDL-C, Low-density lipoprotein cholesterol; HDL-C, High-density lipoprotein cholesterol
(DOCX)

S2 Table List of stop codon mutations fulfilling the criteria with the exception of association with hs-GH. Abbreviations: MAF, minor allele frequency. P is for association with hs-GH in a linear regression model adjusted for sex and age. Carriers—number of homozygous carriers for minor allele, heterozygous and homozygous carriers for major allele respectively. Mutation shows the effect of the mutation on the transcript.
(XLSX)

S3 Table Mediation analysis of nominally significant traits. One linear regression model is executed per trait with the trait in question as the dependent variable and the stop codon mutation, hs-GH, sex and age as independent variables. The natural logarithms of the traits and of hs-GH are standardized separately in men and women. Abbreviations as previously mentioned.
(DOCX)

S1 Fig Illumina cluster plot for rs35699176. Cluster plot showing genotyping for snp in *ZNF77*. NB, samples with black dots did not pass our quality criteria for genotyping and were thus excluded from the study.
(TIFF)

S2 Fig Illumina cluster plot for rs121909305. Cluster plot showing genotyping for snp in *MYO1A*.
(TIFF)
