# Supplementary material for: Analysis of Low Frequency Protein Truncating Stop-Codon Variants and Fasting Concentration of Growth Hormone
Source: PLoS One. 2015 Jun 18;10(6):e0128348. doi: 10.1371/journal.pone.0128348 (PMC4472854; doi:10.1371/journal.pone.0128348)
Supplement: S1 Table — Abbreviations: LDL-C, Low-density lipoprotein cholesterol; HDL-C, High-density lipoprotein cholesterol (DOCX) [file pone.0128348.s001.docx]

*Analysis of low frequency protein truncating stop-codon variants and fasting concentration of Growth Hormone*

-The Malmö Diet and Cancer Study

Erik Hallengren, MD; Peter Almgren, MSc; Gunnar Engström, MD, PhD; Margaretha Persson, PhD; Olle Melander, MD, PhD.

**Supporting information table S1:** Clinical characteristics of the study population and individuals in which genotyping was not possible due to missing samples or unsuccessful genotyping.

| **Variable** | **Included** | **No** | **Not included** | **no** |
| --- | --- | --- | --- | --- |
| Number of participants | 5451 |  | 652 |  |
| Female (%) | 3187 (58.5) | 5451 | 336 (52.3) | 643 |
| Age, mean (SD), years | 57.5 (5.9) | 5451 | 57.5 (5.9) | 643 |
| Body Mass Index, Mean (SD), kg/m2 | 25.8 (4.0) | 5444 | 26.0 (3.9) | 643 |
| Waist, mean (SD), cm | 84.1 (13.0) | 5443 | 85.2 (12.8) | 643 |
| Bodyfat percentage, mean (SD), % | 27.6 (7.1) | 5434 | 27.0 (7.1) | 643 |
| LDL-C, mean (SD), mmol/L | 4.20 (0.98) | 4800 | 4.16 (1.05) | 568 |
| HDL-C, mean (SD), mmol/L | 1.38 (0.37) | 4874 | 1.35 (0.38) | 576 |
| Height, mean (SD), cm | 169 (9) | 5444 | 169 (9) | 643 |
| Growth Hormone - males, median (IQR), μg/L | 0.11  (0.06-0.33) | 1729 | 0.12 (0.06-0.41) | 225 |
| Growth Hormone - females, median (IQR), μg/L | 1.21  (0.38-3.12) | 2405 | 1.21 (0.39-3.60) | 248 |

Abbreviations: LDL-C, Low-density lipoprotein cholesterol; HDL-C, High-density lipoprotein cholesterol
