# Supplementary material for: Analysis of Low Frequency Protein Truncating Stop-Codon Variants and Fasting Concentration of Growth Hormone
Source: PLoS One. 2015 Jun 18;10(6):e0128348. doi: 10.1371/journal.pone.0128348 (PMC4472854; doi:10.1371/journal.pone.0128348)
Supplement: S3 Table — One linear regression model is executed per trait with the trait in question as the dependent variable and the stop codon mutation, hs-GH, sex and age as independent variables. The natural logarithms of the traits and of hs-GH are standardized separately in men and women. Abbreviations as previously mentioned. (DOCX) [file pone.0128348.s003.docx]

| SNP | Variable | p for hs-GH | p for snp |
| --- | --- | --- | --- |
| [rs35699176](http://www.ncbi.nlm.nih.gov/SNP/snp_ref.cgi?type=rs&rs=rs35699176) | Height | 0.009 | 0.08 |
| [rs121909305](http://www.ncbi.nlm.nih.gov/SNP/snp_ref.cgi?type=rs&rs=rs121909305) | BMI | 9.5858E-56 | 0.73 |
|  | Bodyfat% | 9.062E-51 | 0.66 |
|  | Waist | 2.3472E-58 | 0.87 |
|  | HDL | 1.9086E-15 | 0.08 |

**Supporting information table S3:** Mediation analysis of nominally significant traits.

One linear regression model is executed per variable with the variable in question as the dependent variable and the stop codon mutation, hs-GH, sex and age as independent variables. Abbreviations as previously mentioned.
