# Supplementary figures and images for: Analysis of Low Frequency Protein Truncating Stop-Codon Variants and Fasting Concentration of Growth Hormone
Source: PLoS One. 2015 Jun 18;10(6):e0128348. doi: 10.1371/journal.pone.0128348 (PMC4472854; doi:10.1371/journal.pone.0128348)

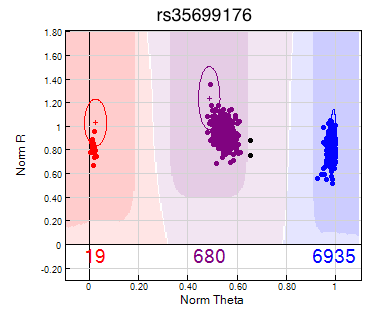

Supplement: S1 Fig — Cluster plot showing genotyping for snp in ZNF77. NB, samples with black dots did not pass our quality criteria for genotyping and were thus excluded from the study. (TIFF) [file pone.0128348.s004.tiff]

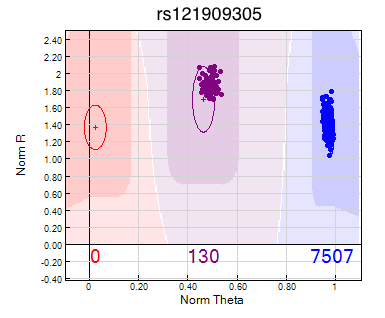

Supplement: S2 Fig — Cluster plot showing genotyping for snp in MYO1A. (TIFF) [file pone.0128348.s005.tiff]
